# Understanding Wellbeing Among College Music Students and Amateur Musicians in Western Switzerland

**DOI:** 10.3389/fpsyg.2019.00820

**Published:** 2019-05-03

**Authors:** Roberta Antonini Philippe, Céline Kosirnik, Noémi Vuichoud, Aaron Williamon, Fabienne Crettaz von Roten

**Affiliations:** ^1^Laboratoire PHASE, Faculté des Sciences Sociales et Politiques, Institut des Sciences du Sport, Université de Lausanne, Lausanne, Switzerland; ^2^Centre for Performance Science, Royal College of Music, London, United Kingdom; ^3^Faculty of Medicine, Imperial College London, London, United Kingdom; ^4^Faculté des Sciences Sociales et Politiques, Institut des Sciences du Sport, Université de Lausanne, Lausanne, Switzerland

**Keywords:** wellbeing, quality of life, health, college music students, amateur musicians, Switzerland

## Abstract

Musical performance requires the ability to master a complex integration of highly specialized motor, cognitive, and perceptual skills developed over years of practice. It often means also being able to deal with considerable pressure within dynamic environments. Consequently, many musicians suffer from health-related problems and report a large number of physical and psychological complaints. Our research aimed to evaluate and analyze the wellbeing of two distinct groups of musicians, college music students and amateur performers in the French-speaking part of Switzerland. A total sample of 126 musicians was recruited for the study (mean age ±*SD* = 22.4 ± 4.5 years, 71 male). Wellbeing was assessed through the World Health Organization Quality of Life-BREF questionnaire evaluating two general measures, quality of life (QoL) and general health, and four specific dimensions: physical health, psychological health, social relationships, and environment. For both groups, respondents’ QoL was high on each measure: median scores were higher than 4 for the two general measures and higher than 70 for the four specific dimensions. Among the dimensions, respondents had the highest mean score for environment (75.0), then social relationships and physical health (74.0 and 73.8, respectively), and finally, psychological health (70.3). Differences between groups of musicians emerged in terms of overall QoL and general health, as well as the physical health dimension, where college music students scored lower than the amateur musicians; conversely, college music students scored higher than the amateurs on social relationships. Our overview of musicians’ wellbeing in Western Switzerland demonstrates that, while music making can offer some health protective effects, there is a need for greater health awareness and promotion among advanced music students. This research offers insight into musicians’ wellbeing and points to the importance of involving different actors (teachers, administrators, support staff) in facilitating healthy music making.

## Introduction

Wellbeing is a major preoccupation for the World Health Organization (WHO), as outlined in the policy program *Health 2020* (Lindert et al., 2015). European member states have agreed a unique measure to assess subjective wellbeing, life satisfaction. Although a universal definition or measure of subjective wellbeing does not exist, “In general, subjective wellbeing refers to a cognitive process of contentment, satisfaction or happiness derived from optimal functioning. Optimal functioning is a relative rather than an absolute concept as the benchmark for judging lives in an individual’s perception of his or her own aspirations" (Lindert et al., 2015, p. 732).

Wellbeing is a multidimensional phenomenon and refers to emotional and cognitive dimensions of subjective experiences resulting from the individual evaluation of several facets of life. Research on wellbeing has revealed two fundamental perspectives (Disabato et al., 2016): hedonia (Diener, 1984) and eudaimonia (Ryff, 1989). The hedonic perspective emphasizes the attainment of pleasure and pain avoidance, focusing mainly on happiness. The eudaimonic perspective encompasses a person’s optimal degree of functioning, focusing on meaning and self-realization (Ryan and Deci, 2001) and dimensions of self-acceptance, autonomy, personal growth, positive relationships, environmental mastery, and goals in life (Ryff, 2014). According to Ryan and Deci (2002), if the fundamental needs of autonomy, relatedness and competence are fulfilled, individuals can experience personal growth and wellbeing.

In the field of music, wellbeing has been investigated in two different ways, with music as a facilitator but also as a disruptor of wellbeing. In terms of facilitation, much research has pointed out its strong and positive impact on people’s lives (Pothoulaki et al., 2012; Västfjäll et al., 2012; Perkins and Williamon, 2014). Boyce-Tillman (2000), for instance, showed that music facilitates creativity and, in this way, promotes wellbeing. Also, singing in groups has been associated with positive wellbeing (Davidson, 2008; Boyce-Tillman, 2014). Evans (2015) demonstrated that music can fulfill the three fundamental needs identified by self-determination theory as necessary to wellbeing. Similarly, Dickinson (2018) has listed mechanisms by which music can influence wellbeing: it can motivate, the rhythm can diminish anxiety, music helps alleviate the effects of some disorders (e.g., obsessive-compulsive disorder and depression), it helps to find a balance between personal life and work, and it builds links and promotes exercise as well as release tension. Croom (2012) demonstrated that music can influence positively the five components of wellbeing outlined in Seligman’s PERMA model: positive emotion, engagement, relationships, meaning, and accomplishment (Seligman, 1998). Using the same model, Ascenso et al. (2018) tested professional musicians on the five PERMA dimensions and found that musicians scored high on all five, suggesting that, even among professionals, music is linked with positive wellbeing.

However, research has also shown that making music can be a disruptor of wellbeing as it is linked with the many challenges that musicians face in their practice. Physical pain is one of the consequences in making music at a high level. Only 26.7% reported that they had never experienced performance-related pain (Kenny and Ackermann, 2015). Furthermore, the large majority of professional musicians have experienced at least one medical problem (86%), with 76% suffering at least once from a severe medical problem (Fishbein et al., 1988). They often experience pain (86%) (Leaver et al., 2011), with some experiencing pain that profoundly impairs their performance (Croom, 2012). Prevalence of musculoskeletal disorders among music students has been reported to be anywhere between 35 to 80% (Zaza, 1998; Cruder et al., 2018), with musicians reporting more pain than other university students (e.g., medical students) (Kok et al., 2013). In a study concerning musicians’ musculoskeletal problems, Chong et al. (1989) highlighted differences between student and amateur musicians who were seen in a clinic for such problems: 36% were students and 18% amateurs. Anxiety and distress during performance have also been studied (Kenny et al., 2014; Osborne et al., 2014; Antonini Philippe and Güsewell, 2016). These factors all pose risks to musicians’ wellbeing (Williamon and Thompson, 2006).

Beyond the fact that music can have an influence, positive and negative, on wellbeing, it has been argued that wellbeing can have an impact on facilitating good performance (Williamon, 2004; Kenny, 2011), and yet, the literature shows that musicians engage poorly in health promoting behaviors. Kenny et al. (2014) conducted a survey of professional musicians in Australia, and the results reveal sub-optimal mental health and poor health behaviors. However, efforts are being made in some countries (e.g., United Kingdom, South Africa, and Australia) to reconfigure music training programs to incorporate insight from health professionals and active health education and literacy (Perkins et al., 2017).

The paradoxical bidirectional relationships between music and wellbeing as outlined above are puzzling and warrant further investigation. No doubt, there is large variation in the instruments and methods used when studying wellbeing, as well as cultural differences between countries and geographical regions. Indeed, many of the existing scales are based on morbidity, mortality, and the impact of disorders or disease on daily activities and behavior on perceived health, containing measures of disabilities (WHO, 1996). They are, as such, problematic as they do not capture QoL and are often culturally influenced depending on where they are developed.

In order to avoid these problems, the World Health Organization Quality of Life (WHOQOL) Group developed a multidimensional scale of QoL linked to their health policy program centered on a humanistic approach (WHOQOL Group, 1991, 1998). The resulting WHOQOL questionnaire focuses on functional and positive aspects and considers the social context as well as the environment in which people live (WHO, 1996). QoL is defined as “an individual’s perception of their position in life, in the context of the culture and value systems in which they live and in relation to their goals, expectations, standards, and concerns. It is a broad ranging concept, affected in a complex way by the person’s physical health, psychological state, level of independence, social relationships and their relationship to salient features of their environment” (WHOQOL Group, 1995, p. 1404). While the concepts of QoL and subjective wellbeing are similar (Diener et al., 1999), measurement of QoL is more widespread than that of subjective wellbeing, with QoL being more specific and commonly found in the methodological and scale development domain (Hawthorne et al., 2006). The Satisfaction with Life Scale (Diener et al., 1985; Pavot and Diener, 1993) is a notable exception and measures subjective wellbeing.

The WHO has developed many alternative measurement tools from the original WHOQOL questionnaire, which was composed of 100 items. For example, the WHOQOL-BREF examines life satisfaction and general health, as well as four component scores of physical health, psychological health, social relationships and environment. This 26-item questionnaire has been used in many studies, some in the field of music (Clift et al., 2007, 2010; Johnson et al., 2013; Garrido et al., 2016; Chang et al., 2018). These studies have focused on the four component scores of the questionnaire, but other studies have used only some dimensions or just the total WHOQOL-BREF score. For example, Dritsakis et al. (2017) investigated the positive effects of music on the wellbeing of patients with cochlear implants in three domains: physical health, psychological health, and social relationships. Mitchell et al. (2007) investigated the effects of music listening on the wellbeing of chronic pain sufferers, and their results revealed a higher total score for patients who listen frequently to music and who perceived it as personally important.

As mentioned before, culture can influence the ways in which people define and characterize wellbeing, and cultural differences also affect the impact of health promotion interventions within a given country or geographical region. If we refer to Switzerland, there seems to be a growing interest in understanding and promoting musicians’ health. In 1997, the Swiss society for music medicine was founded in order to help musicians cope with performance-related physical problems. Recently, they initiated interdisciplinary consultations composed of musicians, doctors, psychologists, and diverse therapists in order to support musicians in their daily work (Berchtold-Neumann, 2018). Such consultations exist in the German- and Italian-speaking parts of Switzerland but not yet in the French-speaking region. Only a few Swiss studies have investigated the impact of music on the general population. One study by Thoma et al. (2012), conducted in Zurich, focused on the impact of music listening on emotion regulation and stress reactivity, as well as physiological and psychological functioning. Among other measures, they used the WHOQOL-5, a five-item version of the WHOQOL to test musicians’ life satisfaction, but the psychometric properties of the questionnaire have not yet been validated. Another Swiss study was conducted on the influence of attending cultural and arts events on wellbeing. This longitudinal study with Swiss population aged 14 years and older (engaged actively or passively in cultural activities such as playing an instrument or singing, painting, sculpting) provided little evidence of a causal influence (Weziak-Białowolska, 2016). In fact, results showed that long-term health and wellbeing did not improve significantly as a result of any specific activity in the cultural arena.

Only a few studies have been devoted to the understanding of health and QoL among musicians during their formative years in conservatoire training (Williamon and Thompson, 2006; Kreutz et al., 2009). Therefore, our research aimed to evaluate and analyze the wellbeing of college music students compared with amateur musicians in the French-speaking part of Switzerland. This permits direct comparisons between these two groups, as well as the exploration of differences between those who take part in judged performances (e.g., competitions) and between men and women.

## Materials and Methods

### Respondents

One hundred and thirty instrumental and vocal musicians and singers took part in the study, recruited via different music schools and music colleges by contacting directors, teachers and, also, via personal contacts. Four musicians were excluded from analyses because they performed computer-assisted music but did not play an instrument such as those found in a symphony orchestra, leaving a final sample of 126 respondents (mean age ±*SD* = 22.4 ± 4.5 years, 71 male) of two groups: HEM participants (“HEM” being, in French, “Haute Ecole de Musique”) and non-HEM participants (i.e., amateur musicians) serving as the comparison group, as detailed in Table 1. A sample size of 126 is above the number required to calculate independent samples *t*-tests with a medium effect size [Cohen (1992), *ES* = 0.5], a first error alpha of 0.05 and a power of 0.8, corresponding to 104 according to Gpower (version 3.1).

**Table 1:** Characteristics of music college students (HEM) and amateur musicians (non-HEM) in the sample, including *n* (%) for categorical variables, mean (SD) for continuous variable, and Chi-square tests of independence.

	Total	HEM	Non-HEM	Chi-square test
*Characteristic*	*n* = 126	*n = 46*	*n = 80*	
Sex (male)	71 (56.3%)	25 (54.3%)	46 (57.5%)	0.118
Age	22.4 (4.5)	23 (3.0)	22 (5.2)	25.30*
≤20	54 (42.9%)	7 (15.2%)	47 (58.8%)	
21–24	40 (31.7%)	25 (54.3%)	15 (18.8%)	
≥25	32 (25.4%)	14 (30.4%)	18 (22.5%)	
*Musical instrument*				30.62*
Wind	46 (36.5%)	30 (65.2%)	16 (20.0%)	
Strings	18 (14.4%)	3 (6.5%)	15 (18.8%)	
Keyboard	30 (23.8%)	6 (13.0%)	24 (30.0%)	
Percussion	18 (14.3%)	1 (2.2%)	17 (21.3%)	
Voice	14 (11.1%)	6 (13.0%)	8 (10.0%)	
*Judged performance* (Yes)	96 (76.2%)	45 (97.8%)	51 (64.6%)	18.06*


*^∗^*p* < 0.05.*

### Procedure and Methods

All musicians provided socio-demographic and music-related information before completing the WHOQOL-BREF questionnaire. Sociodemographic variables included sex and age. Age was recoded into three groups: ≤20 years, 21–24 years, and ≥ 25 years, using the higher group as the reference category. Data on performance specialism and music education was also collected. For specialism, musicians reported their instrument, and five categories were created: wind, strings, keyboard, percussion, and voice. Participants then indicated their music educational status: college music students seeking Bachelor or Master qualifications (i.e., HEM) or amateur musicians (i.e., non-HEM), and whether or not they participate in judged performances or competitions (Yes or No).

The majority of respondents completed the paper version (*n* = 117), but some completed the questionnaire online, with access provided by email. Information about the study was given to all participants and highlighted the confidentiality and anonymity of their participation. Musicians participated voluntarily and could stop answering the questionnaire at any time. Written informed consent was obtained from all participants.

The WHOQOL-BREF questionnaire, which consists of 26 items, was used to measure musicians’ QoL. This version of the original 100-item WHO (1991) instrument has been adapted in various languages, including French, as used in this study. The WHOQOL-BREF is a self-administrated instrument that assesses general wellbeing. It consists, firstly, of two general items: overall QoL (rated from 1 *Very poor* to 5 *Very good*) and general health (from 1 *Very dissatisfied* to 5 *Very satisfied*). It then progresses to a series of 24 items on four QoL dimensions: physical health (7 items), psychological health (6 items), social relationships (3 items), and environment (8 items). Each item is scored on a 5-point intensity scale (1 *Not at all* to 5 *Extremely*), a 5-point evaluation scale (1 *Very dissatisfied* to 5 *Very satisfied*), a 5-point capacity scale (1 *Very poor* to 5 *Very good*), and a 5-point frequency scale (1 *Never* to 5 *Always*).

The physical health dimension includes questions on daily activities; dependence on medicinal substances and medical aids; energy and fatigue; mobility; pain and discomfort; sleep and rest; and work capacity. The psychological health dimension assesses knowledge of bodily image and appearance; negative feelings; positive feelings; self-esteem; spirituality/religion/personal beliefs; and thinking, learning, memory, and concentration. Social relationships are characterized through personal relationships; social support; and sexual activity. Finally, environment is captured through questions on financial resources; freedom, physical safety, and security; health and social care; accessibility and quality; home environment; opportunities for acquiring new information and skills; participation in and opportunities for recreation/leisure activities; physical environment (pollution/noise/traffic/climate); and transport.

The dimension’s mean scores were calculated according to the procedure described by the WHO (1996) with a transformation to a 0–100 scale. Scores are scaled in a positive direction, with a higher score corresponding to higher QoL. The reliability coefficients of the four dimensions were, respectively, Cronbach alpha = 0.68, 0.75, 0.64, and 0.74, which indicate minimally acceptable reliability for dimensions one and three, and respectable reliability for dimensions two and four (De Vellis, 2003).

This study was led by the sport psychology laboratory at the Institute of Sport Sciences, University of Lausanne, and ethical approval was granted by the *Commission cantonale d’éthique de la recherche sur l’être humain* (CER-VD). Authorization to use the WHOQOL-BREF was granted by the WHO through a standard user-agreement form.

### Data Analyses

Descriptive statistics were calculated for each variable. Chi-square independence tests were performed to assess differences among HEM and non-HEM respondents in terms of sex, age group, musical instrument, and participation in judged performances. In comparisons of groups (e.g., sex, age groups) among the four QoL dimensions, MANOVAs were used. For comparing HEM vs. non-HEM, we used sex and age group as covariates whereas for comparing participants vs. non-participants in judged performances, we used sex, age group and education as covariates. Homogeneity of the matrix of variance-covariance was assessed with Box’s *M*-test and normality with the Shapiro–Wilk test. When the MANOVA was significant, we performed univariate *F*-tests with a significance correction. We also calculated effect sizes (Cohen, 1992). Finally, a multivariate logistic regression was performed to compare the HEM and non-HEM groups on the four QoL dimensions after controlling for sex and age; the model meets Vittinghoff and McCulloch (2007) rule of 5 – 9 outcome events per predictor variable. The level of statistical significance was set at 0.05. All analyses were performed using SPSS (version 25).

## Results

### Descriptive Characteristics of the Study Sample

Within the sample, 56% of respondents were men and 43% women (Table 1). In musical terms, most played wind instruments (37%), followed next by keyboard (24%), and 76% had participated in judged performances or competitions. A third of respondents followed HEM education.

Table 1 displays the descriptive statistics for HEM and non-HEM respondents. The distribution of age, musical instrument, and participation in judged performances varied significantly between HEM and non-HEM respondents: HEM musicians were more frequently in the higher age groups, they played more frequently wind instruments, and participated more frequently in adjudicated performances.

### Wellbeing

The QoL of participants was high on each measure: median scores were higher than 4 for the first two measures and higher than 70 for the four QoL dimensions (Table 2). Among the dimensions, respondents had the highest mean score for environment (75.0), then on social relationships and physical health (74.0 and 73.8, respectively) and, finally, on psychological health (70.3).

**Table 2:** Descriptive statistics for wellbeing, including overall QoL, general health, and each of the four dimensions of the WHOQOL-BREF (WHO, 1991).

	M (SD)	Median	Min	Max
Overall QoL	4.35 (0.65)	4	2	5
General health	4.09 (0.85)	4	2	5
**WHOQOL-BREF**				
Physical health	73.81 (13.14)	75.0	32	100
Psychological health	70.34 (14.35)	71.0	29	100
Social relationships	73.99 (17.37)	75.0	8	100
Environment	75.00 (13.33)	75.0	31	100


Quality of Life varied little among sociodemographic groups. There was no significant relationship between each of the two general measures and sex (respectively, $\chi_{2}^{2}$ = 2.96, *p* = 0.227, and $\chi_{2}^{2}$ = 0.201, *p* = 0.904), nor for age groups ($\chi_{4}^{2}$ = 5.19, *p* = 0.269, and $\chi_{4}^{2}$ = 1.79, *p* = 0.775).

The four QoL dimensions did not differ according to sex (Pillai = 1.265, *p* = 0.288, *Eta^2^* = 0.040). Nevertheless, the Psychological health dimension differed significantly as a function of sex (*F*_1,124_ = 4.05, *p* = 0.046, *Eta^2^* = 0.032), where female musicians had lower scores, *M(SD)* = 67.4 (13.5), than male musicians, *M(SD)* = 72.6 (14.69). The four QoL dimensions did not differ according to age group (Pillai = 0.714, *p* = 0.679, *Eta^2^* = 0.023).

### Relationships Between Wellbeing Factors and Education, Judged Performances, and Sex

The two general measures of QoL were each significantly related to education. For overall QoL, a lower percentage of HEM respondents answered “very good” compared with non-HEM respondents (26% vs. 54%), $\chi_{2}^{2}$ = 9.11, *p* = 0.011, and for general health, fewer HEM respondents answered “very satisfied” (22% vs. 41%), $\chi_{2}^{2}$ = 10.27, *p* = 0.006.

The four QoL dimensions were also related to education, with sex and age group as covariates (Pillai = 4.785, *p* = 0.001, *Eta^2^* = 0.140, with the two covariates not reaching significance). The QoL dimensions showed few significant differences (Table 3). The physical health dimension varied significantly by education (*p* = 0.000 and *Eta^2^* = 0.098): HEM respondents had a lower QoL physical score than non-HEM respondents (*M* = 69.46 vs. 76.31).

**Table 3:** Comparisons of the four WHOQOL-BREF dimensions between music students (HEM) and amateur musicians (non-HEM).

	HEM (*n* = 46)		Non-HEM (*n* = 80)				Effect size
	*M*	*SD*	*M*	*SD*	*F*_1,121_	*p*	*Eta^2^*
Physical health	69.46	12.79	76.31	12.75	13.159	0.000*	0.098
Psychological health	69.09	12.54	71.06	15.32	1.713	0.193	0.014
Social relationships	77.02	17.19	72.25	17.35	0.932	0.336	0.008
Environment	72.83	15.64	76.25	11.73	3.131	0.079	0.025


*^∗^significant.*

The two general measures of QoL were not related with participation in judged performances: overall QoL ($\chi_{2}^{2}$ = 4.49, *p* = 0.109) and general health ($\chi_{2}^{2}$ = 1.20, *p* = 0.549). The four QoL dimensions were related to taking part in judged performances, with sex, age group and education as covariates (Pillai = 3.47, *p* = 0.010, *Eta^2^* = 0.107, with education the only covariate reaching significance *p* = 0.000). The QoL dimensions showed few significant differences. Psychological health varied significantly according to judged performance as did Environment (see Table 4 respectively, *p* = 0.015, *Eta^2^* = 0.049; *p* = 0.035, *Eta*^2^ = 0.037) where respondents doing more judged performances had higher scores than those who did not.

**Table 4:** Comparisons of the four WHOQOL-BREF dimensions between musicians who take part in judged performances and those who do not.

	Not judged (*n* = 29)		Judged (*n* = 96)				Effect size
	*M*	*SD*	*M*	*SD*	*F*_1,119_	*p*	*Eta^2^*
Physical health	72.55	14.39	74.22	12.86	1.955	0.165	0.016
Psychological health	64.62	15.70	71.84	13.45	6.123	0.015*	0.049
Social relationships	73.45	14.94	74.22	18.18	0.730	0.394	0.006
Environment	71.69	13.15	75.91	13.34	4.559	0.035*	0.037


*^∗^significant at *p* < 0.05.*

We also performed a multivariate logistic regression comparing HEM and non-HEM respondents on the four QoL dimensions after controlling for sex and age. Model 2 explained the data well, with a Nagelkerke *R*^2^ coefficient (i.e., a measure of the strength of the relationship) of 0.42 and 77% of the respondents correctly classified. Overall, three factors were significant: age group (*z*_2_ = 22.48, *p* = 0.000), physical health (*z*_1_ = 9.83, *p* = 0.002), and social relationships *z*_1_ = 5.31, *p* = 0.021) (Table 5). What distinguishes HEM and non-HEM musicians? Adding 1 to the physical health dimension score decreased the odds of being in the HEM group (odd ratios from 1 to 0.919), while conversely, adding 1 to social relationships dimension score increased the odds of being in the HEM group (odds ratio from 1 to 1.038).

**Table 5:** Multivariate logistic regression on the four WHOQOL-BREF dimensions controlling for sex and age.

	Estimate	Std. error	Exp (B)	Wald stat.	*p*
**MODEL 1**		
Sex	–0.13	0.47	0.87	0.08	0.773
Age group				21.85	0.000*
=20	–1.67	0.54	0.19	9.53	0.002*
21–24	0.76	0.48	2.14	2.46	0.116
**MODEL 2**		
Sex	0.13	0.47	1.14	0.08	0.773
Age group				22.48	0.000*
≤20	–1.97	0.63	0.14	9.87	0.002*
21–24	0.93	0.54	2.53	2.95	0.086
Physical health	–0.08	0.03	0.92	9.82	0.002*
Psychological	0.001	0.02	1.00	0.004	0.947
Social relationships	0.04	0.02	1.04	5.31	0.021*
Environment	0.01	0.02	1.01	0.08	0.771


*^∗^significant at *p* < 0.05.*

## Discussion

The QoL of music college students (i.e., Bachelor and Master) and amateur performers who took part in this study was high, mirroring the positive wellbeing profiles reported by Ascenso et al. (2018) in their study of professional musicians. Median scores for overall QoL and general health were both higher than 4, and on the four QoL dimensions, they were higher than 70. Nonetheless, the study highlights some intriguing differences between college music students and amateur musicians, for instance with amateur musicians scoring significantly higher than music students on overall QoL and general health.

### Overall Quality of Life

When comparing music college students with amateur musicians, our results showed that music college students evaluated their overall QoL more negatively than amateur musicians, but this was not related to participation in judged performances and competitions.

As defined by the WHO (1996), QoL includes many domains of functioning. This subjective perception is affected by personal physical health, psychological states, social relationships, and environmental features (Saxena and Orley, 1997).

Our findings underline the results presented in Kreutz et al.’s (2009) study suggesting that music performance students tend to neglect health promoting behaviors (e.g., stress management, physical activity). Our results do not consider health promoting behaviors but highlight the fact that QoL among music college students seems to be influenced negatively by physical and psychological factors (Kreutz et al., 2009). These results can partially be explained by the unique educational context of music colleges, which generates both physical and psychological challenges to health. For example, Kenny (2004) showed that musicians at different stages of their careers, report different sources of stress. The top four sources among professional musicians were separation from family, irregular working hours, monotony of rehearsals, and traveling. By contrast, the top four stressors for student musicians were uncertainty about future employment, professional auditions, backstabbing, and irregular working hours (Steptoe, 1989). Therefore, our results are not surprising, as music college students face many difficulties that pose consequences for their overall QoL.

However, the unique educational context of music colleges can also present opportunities (Perkins et al., 2017). Indeed, support sources for health and wellbeing within conservatoires, including improved access to health professionals and welfare staff and specific health promotion initiatives, are now being developed (Perkins et al., 2017). In the French-speaking part of Switzerland, different initiatives are implemented to help music students in their daily work. For example, courses and training to manage stress during judged performances are offered to students, but these courses are often optional. And, as mentioned earlier, this region of Switzerland is less developed in this respect than the German- and Italian-speaking parts, which offer interdisciplinary consultations composed of musicians, doctors, psychologists, and other therapists (Berchtold-Neumann, 2018).

### General Health

Our results also show that music students evaluated their general health more negatively than amateur musicians, unrelated to participation in judged performances, and competitions.

This matches findings among music students reported by Araújo et al. (2017) emphasizing that injury and ill-health among musicians are frequent and well documented in the literature. These issues mainly concern physical problems and suffering (Zander et al., 2010; Bonde et al., 2018). Also, as found in different studies (Ginsborg et al., 2009; Kreutz et al., 2009), Panebianco-Warrens et al. (2014) highlighted the fact that musicians have poor health habits especially concerning physical activity, stress management and nutrition. However, this concept of health is linked to physical aspects as well as to psychological states, for example coping with stress, dealing with negative feelings, and emotions. Williamon (2004) has underlined how physical activity can optimize musicians’ skills by enhancing their physiological and psychological responses to performances (p. 163). However, a study comparing music performance students with non-music performance students revealed that musicians do not seem to engage in such activity and tend to have a less healthy lifestyle overall (Ginsborg et al., 2009).

Our results suggest that, as music college students’ lives are centered on music and performance, these musicians’ main focus may inhibit the importance they give to promoting their general health through physical activity, nutrition education or stress management training.

### Physical Health

Concerning the physical health dimension, our results show that amateur musicians report better physical health than college music students. This is not surprising, as it is well known that music students frequently report pain or discomfort linked to bad posture, excessive practice on their instrument, and performance anxiety (Williamon and Thompson, 2006). The physical health score could be influenced by the pain and discomfort subscale of the WHOQOL-BREF. Pain and discomfort can be caused by performance-related injuries: muscle and tendon injuries, joint issues, nerve compression disorders, and central nervous system disorders. Moreover, the risk of injury increases with increased hours of practice (Kenny and Ackermann, 2009).

Sleep and rest, energy, and fatigue are also evaluated through different items. As some studies have shown that musicians report high levels of exhaustion, stomachaches, headaches, sleep disturbances (Kenny, 2004) and irregular sleep schedules (Araújo et al., 2017; Pecen et al., 2018), these sub-themes could impact on the physical health mean score.

Finally, several studies have highlighted that musicians tend to use drugs or substances: drinking alcohol (Kenny et al., 2014), using beta-blockers (Fishbein et al., 1988) and other prescribed medication (e.g., antidepressants and tranquilizers) or even illicit drugs (e.g., amphetamines, cannabis, cocaine, ecstasy, hallucinogens, and opiate) (West, 2004). Dependence on medicinal substances and medical aids’ subscale could be a strong item influencing musicians’ physical health.

Psychological factors may be involved in the genesis or maintenance of physical problems (Spahn et al., 2001). One study of musicians reported that psychosomatic aspects play a decisive role in musicians’ somatic problems and that these should be addressed in treatment to avoid unwarranted medical interventions (Kenny and Ackermann, 2009).

Our results do not specify which aspect (e.g., fatigue, injuries, and use of substances) of physical health influences musicians the most. However, it clearly highlights the need for action to empower music college students in taking care of their physical health.

### Psychological Health

Our results indicate higher psychological wellbeing among musicians who take part in judged performances and competitions and lower levels for female musicians when psychological health was examined as a function of sex.

Numerous studies have highlighted the undue psychological pressures of working in music (Kenny and Osborne, 2006; Seinfeld et al., 2013; Pecen et al., 2016). Therefore, our results seem counterintuitive. The finding that musicians who are confronted with judged performance situations have higher psychological wellbeing could be influenced by the thinking, learning, memory, and concentration subscale analyzed through the questionnaire. Musicians often seek perfection (Pecen et al., 2016; Araújo et al., 2017) and, to reach the highest levels of practice, they have to develop working strategies to enhance their performances. Also, musicians have a tendency to feel anxiety and stress when performing (Kenny, 2004; Nielsen et al., 2017; Wijsman and Ackermann, 2018), but the WHOQOL-BREF does not evaluate this aspect. However, as Biasutti and Concina (2014) showed, anxiety is also negatively correlated with experience, practice hours, and coping strategies. As college music students often practice more than amateurs, we could assume that they would be more prepared to play during judged performances. Therefore, musicians confronted with judged performances may develop strategies to face specific difficulties (Kaspersen and Goetestam, 2002) and seek help to be prepared (Williamon, 2004). These arguments could partially explain the present findings.

According to our results, female musicians present lower psychological health scores. This result is in line with previous large-scale normative studies using the WHOQOL-BREF, which report significantly lower means for women compared with men (Skevington et al., 2004). This psychological dimension score may be influenced by the self-esteem, body image, and negative and positive feelings sub-themes.

During the past two decades, a large number of studies have examined sex differences in self-esteem (Twenge and Campbell, 2001; Orth et al., 2010, 2012; Shaw et al., 2010). Researchers report that, at every age, men tend to have a higher level of self-esteem than women worldwide (Bleidorn et al., 2016). But, how can we explain this tendency? The concept of self-esteem has been investigated through the influence of sex-specific body satisfaction (Lerner et al., 1973, 1976). Body image is frequently linked to self-esteem as the evaluation of physical appearance is subjective and can either be positive or negative (Forrest and Stuhldreher, 2007).

Body image or physical appearance has been established as an important aspect of wellbeing. Physical appearance self-evaluation (i.e., body-esteem) is a specific domain of self-esteem, especially studied in female populations, showing that body esteem is a construct contained within the hierarchical framework of global self-esteem (Seo and Son, 2014). This construct emphasizes the person’s affective evaluation of the body and feelings associated with personal body image. In the elite sporting context, different researchers have reported greater body dissatisfaction among women due to idealized shape or distorted and dissatisfied subjective body images (Smolak et al., 2000; Ferrand et al., 2005; de Bruin et al., 2007).

Finally, findings from different studies have repeatedly shown a higher prevalence of anxiety and depression diagnoses in women compared with men (Rae and McCambridge, 2004; Ryan, 2004; Yondem, 2007). A study conducted with singers showed that female musicians reported higher work demands and higher stress symptoms than their male colleagues (Holst et al., 2012). Female performers are a higher-risk group and more likely to need specific help. These results have been linked to the differences on how men and women respond to stress and the use of coping strategies (Barlow, 2001; Craske, 2003; Hammen, 2005). It is also crucial to take this aspect into account as positive and negative feelings are often observed to play an important role in health promoting behaviors (Bandura, 1997; Kreutz et al., 2009).

### Social Relationships and Support

Finally, our results highlight that social relationships and social support increase the chance of reaching a high level in the field of music.

In a recent study conducted by Ascenso et al. (2017), musicians highlighted the importance of family, social and work-related connections to ensure positive functioning. Also, practicing music in groups seems to enhance positive social relationship (Clift et al., 2007, 2010; Ascenso et al., 2018). Some musicians even consider chamber and orchestral groups as part of their families, generating a group identity and positive feelings (Ascenso et al., 2017). However, Cooper and Wills (1989) have highlighted tense relationships between colleagues within music institutions, causing stress.

In contrast to musical groups, solo-oriented musicians often face isolation and loneliness. Therefore, they have to find other ways of establishing and maintaining social relationships (Ascenso et al., 2017), for example creating new social circles outside the music community through different activities. In the field of sport, it is well established that good social support generates higher levels of performance and wellbeing, especially among young athletes. Coach-, parent- and peer-support play an important role in enhancing athletes’ motivation (Sheridan et al., 2014). Reis and Gable (2003) also highlighted the importance of strong relationships among the general population. It seems that this social dimension is central to musicians’ wellbeing (Ascenso et al., 2017) and, as our results suggest, should be encouraged.

### Future Research

Several additional directions should be taken into account in future research. First, our sample was not representative of Switzerland’s musician population, nor wholly representative of the French-speaking part of Switzerland. The sample did not include students from all conservatories and music schools from the region. Regional culture is assumed to be similar across the French-speaking part of Switzerland; nevertheless, we could not determine the influence of musicians’ personal cultural backgrounds on their health and wellbeing evaluations (Steptoe and Wardle, 2001; Wardle et al., 2004; Jylhä, 2009). Second, some external parameters that could have influenced musicians’ answers and evaluations of their wellbeing require greater control. We did not know, for instance, the full extent to which each participating musician was individually exposed to health education initiatives, in their training or their personal lives. Also, participants answered the questionnaire at one specific moment, and we did not control for whether it was a particularly busy period (e.g., examinations, auditions) or a calm period. Third, our results are based on self-reports and could have been flavored by social desirability, stressful or difficult periods during which they filled in the questionnaire (e.g., injuries, personal issues), or career aspirations that could possibly have influenced their answers. Fourth, replication of the study with a larger sample would provide strong support for these findings and is therefore a task for future research. Finally, subsequent studies should investigate differences in wellbeing and health habits between classical, jazz, pop and rock musicians. Indeed, these musical styles convey different philosophies that could impact musicians’ health attitudes, perceptions and behaviors.

### Conclusions and Practical Implications

This research offers important insight into musicians’ health and has implications for the future about the extent of health education programs in music education settings. Our overview of musicians’ wellbeing in the French-speaking part of Switzerland underlines the importance of helping musicians to be aware of their health in order to take care of themselves. As Ascenso et al. (2017) said, “a clear sense of self appears as an overarching sustainer of wellbeing” (p. 65). Therefore, it is crucial to empower aspiring young musicians and accompany them in the process of health and wellbeing promotion. However, different specialists should be involved in ensuring musicians’ health and wellbeing, not only their teachers and peers (Williamon, 2004; Williamon and Thompson, 2006; Williamon et al., 2017). Physical care has to be administered by professionals, specialized with musicians, ensuring their postural quality and overall musculoskeletal health. With regard to psychological health, musicians should have access to psychologists and counselors in case of clinical problems and performance coaches and psychologists to enhance their performance. Also, they could benefit from the help of, for example, relaxation therapists or hypnotherapists. Concerning social relationships and support, musicians should have the possibility to plan mediation with people concerned when facing interpersonal difficulties. However, it also falls on institutional structures (through administrators, teachers, and so on) to ensure possibilities for good social relations within places of work and study. Finally, regarding the environment, music institutions should provide suitable working conditions and easy access to training rooms.

Worldwide, efforts are being made to propose health education programs for college music students. Some authors, such as Braden et al. (2015), have already highlighted the positive impact of health and psychological skills enhancement programs within music school curricula (Matei et al., 2018). However, in Switzerland, more efforts could be invested in this regard.

The present study is only a beginning. As Ascenso et al. (2017) point out, a better understanding of the processes underpinning musicians’ wellbeing and QoL are needed, both at a physical and a psychological level. This includes the influence of sleep, the use of substances and fatigue on the health of musicians, as well as the influence of self-esteem, body image, concentration, learning, and memory. Music making is great for health and wellbeing, but for those who commit to music professionally, more action is needed to support their health, both by musicians themselves as well as their teachers, administrators, and support staff.

## Nomenclature

### Resource Identification Initiative

HEM: Haute École de Musique.

WHO: World Health Organization.

WHOQOL: World Health Organization Quality of Life.

QoL: Quality of Life.

## Author Contributions

RAP and CK contributed to the conception and design of the study. RAP, CK, NV, and FCvR organized the database. FCvR performed the statistical analysis. RAP, CK, NV, AW, and FCvR co-wrote the manuscript. All authors contributed to the manuscript revision, read, and approved the submitted version.

## Conflict of Interest Statement

The authors declare that the research was conducted in the absence of any commercial or financial relationships that could be construed as a potential conflict of interest.
